# Surgical Journeys

**Published:** 1990-12

**Authors:** 


### Book Reviews

**SURGICAL JOURNEYS**
**A history of the Surgical Union which became the Surgical Travelling Club of Great Britain.**
**Peter Boreham**
Merlin Books £6.95

In 1921 Sir Berkeley Moynihan invited 28 surgeons to come to Leeds with the intention of forming a club of members 'in practice not associated with teaching hospitals'. In 1909 he had founded a similar club to unite surgeons practising in provincial teaching hospitals and the success of this club encouraged him in the new venture. These two Moynihan foundations may have been the first travelling clubs and there are now several surgical clubs and a number of clubs belonging to other disciplines. These seem to be a peculiarly British institution, does this say something about our 'clubbability' and capacity for friendship? First and foremost they provide an opportunity for travel aborad and seeing at first hand not only the specialist practice but also the political aspect of health provision. They undoubtedly do a great deal to promote friendship amongst doctors, discussion of problems and sharing of knowledge. Peter Boreham has done a great service to his club in accepting the enormous task of going through all their records and producing a highly readable account of their doings. However it has a much wider interest, it is a historical document an account of what surgeons were actually doing in many countries for more than half a century and not by the surgeons themselves but by colleagues who did not necessarily agree with what they saw. It is a mine for the historian and reminder of how far we have come in those years. It is a shock to read that speaking of the period within his memory Sir Clifford Albutt writing to Moynihan in 1924 said 'in those days the staff operated as a whole, all putting their dirty fingers into interesting wounds, and exhaling vapours from their unwashed woollen dressing gowns'. The book is primarily an archive but there are many titbits to be found by anyone who cares to delve into it. The account of a visit to Warsaw in 1977 is full of interest—in spite of communism a certain amount of private practice went on, the commonest operation being to remove tattoed numbers from the arms of former inmates of German concentrations camps. It is a faithful record of the times, not a book to read through but one to possess and with the aid of the comprehensive index to search for information about people and places and always to be rewarded with the unexpected.

